# A Repeated Measures Experiment of Green Exercise to Improve Self-Esteem in UK School Children

**DOI:** 10.1371/journal.pone.0069176

**Published:** 2013-07-24

**Authors:** Katharine Reed, Carly Wood, Jo Barton, Jules N. Pretty, Daniel Cohen, Gavin R. H. Sandercock

**Affiliations:** 1 School of Biological Sciences, University of Essex, Colchester, Essex, United Kingdom; 2 Instituto de Investigaciones, Escuela de Medicina, Universidad de Santander, Bucaramanga, Santander, Colombia; Universidad Europea de Madrid, Spain

## Abstract

Exercising in natural, green environments creates greater improvements in adult's self-esteem than exercise undertaken in urban or indoor settings. No comparable data are available for children. The aim of this study was to determine whether so called ‘green exercise’ affected changes in self-esteem; enjoyment and perceived exertion in children differently to urban exercise. We assessed cardiorespiratory fitness (20 m shuttle-run) and self-reported physical activity (PAQ-A) in 11 and 12 year olds (*n* = 75). Each pupil completed two 1.5 mile timed runs, one in an urban and another in a rural environment. Trials were completed one week apart during scheduled physical education lessons allocated using a repeated measures design. Self-esteem was measured before and after each trial, ratings of perceived exertion (RPE) and enjoyment were assessed after completing each trial. We found a significant main effect (*F* (1,74), = 12.2, *p*<0.001), for the increase in self-esteem following exercise but there was no condition by exercise interaction (*F* (1,74), = 0.13, *p* = 0.72). There were no significant differences in perceived exertion or enjoyment between conditions. There was a negative correlation (*r* = −0.26, *p* = 0.04) between habitual physical activity and RPE during the control condition, which was not evident in the green exercise condition (*r = *−0.07, *p* = 0.55). Contrary to previous studies in adults, green exercise did not produce significantly greater increases in self-esteem than the urban exercise condition. Green exercise was enjoyed more equally by children with differing levels of habitual physical activity and has the potential to engage less active children in exercise.

## Introduction

The prevalence of mental ill health in UK children is rising; approximately 1 in 10 young people suffer from a diagnosable mental health disorder each year [1]. Self-esteem is one indicator of mental health and is defined as: ‘a person’s positive or negative attitude towards the self in totality' [2]. Low self-esteem is a common occurrence in many forms of mental illness [3]; thus methods of improving self-esteem in children are important for mental health.

The positive relationship between exercise and mental health is widely evidenced [4], [5], with a moderate effect size (*d* = 0.51) for changes in children's self-esteem due to exercise [6]. However, approximately 23–34% of males and 35–53% of females aged 11–15 years are failing to meet the daily recommendation of sixty minutes of moderate to vigorous physical activity [7]. Evidence also shows that exposure to nature can improve mental well-being in children; improving cognitive functioning and concentration and reducing psychological distress [8]–[9]. In adults, ‘Green Exercise’ research suggests a synergistic health benefit for self-esteem of engaging in ‘physical activities in the presence of nature’ [10]–[12]. Thus, ‘Green Exercise’ might also be effective at improving self-esteem in children.

A multi-study analysis of green exercise in adults (ten studies, n = 1252) demonstrated significant improvements in self-esteem across many social groups, types of activity and green space [10]. The review also showed a u-shaped ‘dose-response curve’ indicating that even short engagements with nature can have a positive impact. Activities of light intensity (walking, fishing etc) had a greater effect on self-esteem than vigorous activities (mountain biking, conservation activities etc.), although all intensities had positive effects. This suggests that green exercise can complement other approaches in making significant improvements to mental health [10]. While the findings are encouraging, showing a moderate combined effect size (*d* = 0.6), what cannot be determined from these data is if the changes in self-esteem following ‘green exercise’ are greater than those one would expect to observe following another type of exercise intervention.

The benefits of Green Exercise for self-esteem may occur due to enhanced enjoyability of exercise in a natural environment. Green spaces may also encourage greater distractibility from daily stresses, helping people to feel better about themselves [13]. Instead of relying on music or television to provide distraction, findings from green exercise research imply that nature is inherently fascinating and may provide a driver for changes in self-esteem [11]. Outdoor natural environments may also provide a distraction from feelings of fatigue experienced during exercise; thus helping exercise to feel easier [14]. However, all of the above findings are from studies of adults and there are few data regarding the potential additive benefit that nature may have for children when exercise is performed in a green environment [12]. Young people are an important population to study as physical activity habits formed in youth may track into adulthood where adequate levels of activity are protective against many chronic diseases [12].

Much of young peoples' experience of physical activity comes from the physical education (PE) they receive at school [15] and enjoyment of PE is well correlated with children's overall levels of physical activity [16], [17]. Positive experiences gained during PE at school may also promote participation in physical activity during adulthood [18]. One of the aims of PE should be to offer enjoyable experiences that encourage extracurricular activities and promote a lifelong habit of healthy physical activity and experiences likely to create lifelong exercise adherence. Exercising in green environments is associated with reductions in perceptions of exertion [14] and greater enjoyment, satisfaction and positive engagement compared to indoor exercise [19]. Participants' intentions to repeatedly engage in exercise were also greater in the outdoor conditions, implying a better longer-term adherence rate. Therefore, green exercise activities during PE may enhance the self-esteem of children and offer attractive alternatives for children who are typically disengaged with sports and other competitive activities.

The primary aim of this experiment was to determine the effects of green exercise on self-esteem when compared with a control (outdoor, non-green urban area) exercise condition using a counterbalanced, randomised cross-over design. Secondary aims were to determine if there were differences in enjoyment and perceived exertion between exercise environments and how these might be affected by children's levels of fitness and physical activity.

We hypothesised that green exercise would increase self-esteem significantly more than the control condition. Our secondary hypothesis was that children would find green exercise more enjoyable and perception of effort would be lower in comparison to the control condition.

## Methods

### Ethics Statement

The University of Essex faculty ethical review committee approved the study. Permission was granted by the school's head teacher and written parental consent and individual assent was gained for all children who volunteered to participate. Pupils from four year-7 classes took part in the runs, as they were performed as part of a compulsory PE class. However, only children who volunteered and provided consent for participation in the study completed the questionnaires and fitness test. Overall, n = 86 children (11–12 year old) from a local secondary school participated in the study. However due to drop-outs and absences the total number of children completing the study was n = 75.

### Materials

Each child completed the physical activity questionnaire for adolescents to provide an estimate of their habitual physical activity levels [20]. Each participant then completed a version of the 20 m shuttle-run; the FITNESSGRAM PACER test [21] which is a valid method by which to assess aerobic fitness (herein referred to as fitness) in this age group [22].

Immediately before and after each exercise condition, pupils completed the Rosenberg Self Esteem Scale (RSE) [23], reproduced on a single sheet of paper in a large, easy to read font. The RSE is widely used in green exercise research analysing the effects of acute exposure [10]. The instrument provides a self-reported one-dimensional measure of global self-esteem. It consists of 10 statements, each of which are scored on a four point likert scale from strongly agree (1) to strongly disagree (4). An overall self-esteem score is calculated ranging from 10 to 40, with a low self-esteem score representing fewer negative perceptions and thus a higher self-esteem.

Following exercise in both conditions, pupils provided retrospective ratings of perceived exertion using the Ratings of Perceived Exertion (RPE) scale [24]. This scale asks participants to rate how hard they perceive to be working on a scale from 6 (no effort) to 20 (extremely hard) and is designed for use during exercise. RPE was collected immediately at the end of each exercise bout, in order to ensure consistent timing of the scale between the two exercise bouts and so that an overall assessment of each run could be obtained. We also assessed their overall enjoyment of the exercise session using a visual-analogue scale. The scale comprised a line on which participants were asked: ‘*Tell us how much you enjoyed the exercise you just did*’. The line was demarcated at either end with emoticons (sad face and happy face) and the line between emoticons was exactly 12 cm (120 mm). A score between zero and 120 was determined by measuring the exact distance of the mark from zero (sad face) in mm.

### Procedure

We visited the school on three separate occasions. On the initial visit, all pupils were weighed and their stature was assessed. Weight and stature were measured in order to obtain descriptive information about the participants. On the initial visit the study was also explained in full to the pupils. Whilst the study had been explained in detail in the consent form, the initial visit to the school was used to reiterate the key points. Participants were informed about the timings of questionnaires and the importance of completing the questionnaires individually and honestly. Participants were also told that the questionnaires were not being used to evaluate them as individuals and that all data would be anonymised. Furthermore, all pupils were invited to ask for assistance in completing questionnaires if there was anything they did not understand.

Each child was then asked to undertake two bouts of exercise (running) over a standard distance (1.5 miles). We chose this distance so that the exercise would take between 10 and 20 minutes. This exercise duration is effective in promoting positive changes in adult's mental state [25]. The experiment took place in the second week of the new school year (early September) and it was the first PE class of this type undertaken by all pupils. Conditions were randomised and counterbalanced by PE group. Two groups (n = 42) completed the control condition first and two groups (n = 44) completed the green condition first. A total of n = 81 participants completed the green condition and n = 85 completed the control condition; however only 75 participants completed all questionnaires. The runs were conducted at the same time of day, exactly one week apart.

The control condition was a 1.5 mile run of a one lap course around the school campus, on a relatively flat terrain (±2 m). The school is located within a moderate density housing estate on the outskirts of town. The local area has low-to-moderate levels of deprivation; situated at the 59^th^ percentile of area-level deprivation [26] and predominant local land use is classified as urban according to DEFRA (Department for Rural Affairs) standard land-use classification [27].

The school itself is bounded on three sides by housing developments, office buildings and another school. One side of the school grounds is dominated by hard surface playing areas and a multi-use games area or ‘Astroturf’ court. The route ran through the school campus and around the perimeters of the hard surface areas with some time spent on the school playing field, the field itself is, however bounded by housing.

The green condition was also a 1.5 mile run of a single lap course through the local country park. This area was located close to the school and pupils were escorted across the main roads to access the park. The park comprises woodland and dirt paths and the route also ran along the course of a stream. The course had an overall climb (and subsequent descent) of 46m and required participants to get wet feet and jump puddles. At no points in the route could participants see buildings, pylons or other manmade objects except one wooden footbridge used to cross the stream. The park is maintained to a high standard by the local council and volunteers and is largely litter free. In previous work this has been classed as a ‘rural pleasant green environment’ [11].

In each condition, six researchers acted as stewards for both runs, guiding pupils' running direction with hand signals and verbal directions, but they were instructed not to encourage pupils or to provide information on performance or distance. PE staff were present during both conditions and acted as ‘sweepers’ by running behind the last placed pupil to ensure all pupils returned to the start point. Pupils completed the self-esteem scale before each bout of exercise and during the immediate post-exercise period when pupils had returned to the PE department. Following both exercise conditions, we assessed pupils' RPE and enjoyment ratings.

### Statistical analysis

Based on a predicted moderate effect size of *d* = 0.5 [10] and baseline self-esteem measures (Rosenberg Score = 18±4) we determined that the minimum expected changes in self-esteem would require 64 participants at *β* = 0.8, *α* = 0.05. To account for drop outs and non-responders we recruited 86 participants. Overall, 75 participants completed all parts of the procedures providing statistical power of *β* = 0.91, *α* = 0.05.

We created means and standard deviations for all variables and tested between-sex differences using independent t-tests. Where there were significant differences between variables these were analysed separately in further analyses. To determine the effects of green and control exercise on self-esteem we used two-way repeated measures analysis of variance (rm-ANOVA) for green and control exercise with pre- and post-exercise measurements as factors.

We also used repeated measures t-tests, to examine differences in RPE and enjoyment between exercise and green conditions and stepwise multiple regression to assess the relative impact of physical activity, fitness and condition on enjoyment and RPE. We also used Pearson's product moment correlation to assess the relationship measures of physical activity and fitness had with measures of enjoyment and RPE during both conditions.

## Results

The descriptive characteristics of the sample are shown in Table 1 and show similarities in age and stature of boys and girls. As expected, females were significantly heavier than males at this age due to earlier pubertal development. Females reported significantly lower levels of physical activity in the previous seven days and completed fewer laps during the 20 m shuttle-run test indicating lower cardio respiratory fitness compared with males.

**Table 1:** Descriptive anthropometric, physical activity, fitness and self-esteem scores for sample.

	Males		Females		p-value
	Mean	SD	Mean	SD	
Age (years)	11.4	0.2	11.4	0.3	0.98
Stature (cm)	150.2	7.5	148.4	12.0	0.44
Body mass (kg)	40.3	6.5	44.5	10.1	0.03
PAQ score	2.9	0.7	2.5	0.6	0.004
Shuttle run (laps)	67.2	15.3	45.3	15.8	0.001
Self Esteem	17.1	4.0	18.8	4.0	0.09

PAQ-physical activity questionnaire (Kowalski et al. 1997). SE-Self Esteem; measured using the Rosenberg scale (a lower score shows higher self-esteem).

**Figure 1:**
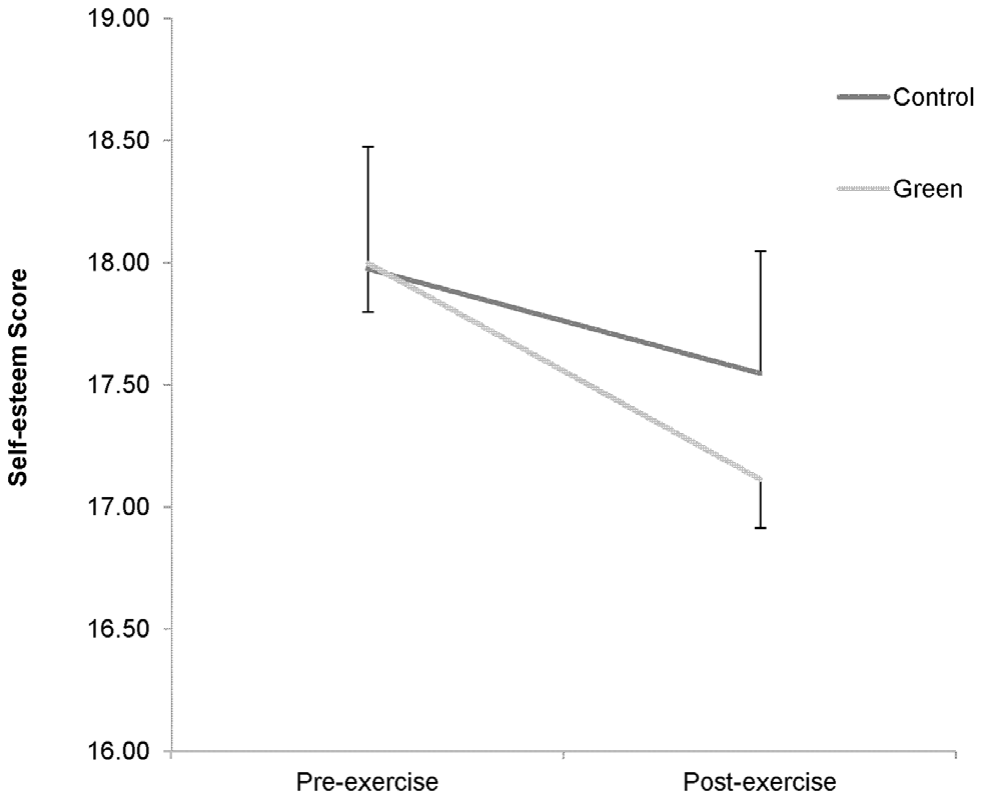
Self esteem pre- and post exercise in control and green exercise conditions. Self esteem; measured using the Rosenberg scale (a lower score shows higher self-esteem).

There were no significant order effects for SE, (*t*(75) = 0.23, *p* = 0.97) enjoyment (*t*(75) = 0.32, *p* = 0.76) or RPE (*t*(75) = 1.4, *p* = 0.15). Figure 1 shows the SE values before and after both exercise conditions (means ± SD). As there were no significant differences in SE between boys and girls or between measurements made before either exercise conditions, male and female cases were analysed together. There was a significant main effect for exercise on self-esteem (*F*(1,74), = 12.2, p<0.001), but no main effect for exercise condition (*F*(1,74) = 0.02, *p* = 0.898) and no interaction (*F*(1,74), = 0.13, *p* = 0.72).

Values for ΔSE, RPE and enjoyment in both conditions are shown in Table 2. RM *t*-tests showed there were no significant differences between green and control exercise in terms of enjoyment (*t*(75)* = *0.43, *p* = 0.66), RPE (*t* (75)* = *0.11, *p* = .914) or change in self-esteem (*t*(75)* = *0.13, *p* = 0.72).

**Table 2:** Changes in self-esteem, ratings of perceived exertion and enjoyment in green exercise versus control.

	Control Exercise		Green Exercise		p-value
	Mean	SD	Mean	SD	
ΔSelf Esteem	0.5	4.2	0.9	2.6	0.72
RPE	13.6	3.6	13.5	3.6	0.91
Enjoyment	83.6	34.7	89.2	31.4	0.66

SE – Self esteem; measured using the Rosenberg scale (a lower score shows higher self-esteem) Δ*Score is pre-test minus post-test value.* RPE – Rating of Perceived Exertion; measured using Borg's 6–20 scale. Enjoyment – Score out of 120 measured using a visual analogue scale (a higher score shows greater enjoyment).

Ratings of perceived exertion and fitness showed similar negative correlations in the control and green exercise conditions (Table 3). There was a significant, negative relationship between physical activity and RPE in the control condition but this relationship was not evident when RPE scores from the green exercise condition were correlated with physical activity (Table 3).

**Table 3:** Relationship of fitness and physical activity with ratings of perceived exertion and enjoyment in the green exercise and control conditions.

		RPE			Enjoyment		
		R value	R^2^ Value	P Value	R value	R^2^ Value	P Value
**Fitness**	Control	−0.37	0.14	0.001	0.45	0.20	0.000
	Green	−0.36	0.13	0.002	0.41	0.17	0.000
**Physical Activity**	Control	−0.26	0.07	0.04	0.30	0.09	0.003
	Green	−0.07	0.00	0.551	0.26	0.70	0.022

RPE – Rating of Perceived Exertion; measured using Borg's 6–20 scale. Enjoyment – Score out of 120 measured using a visual analogue scale (a higher score shows greater enjoyment). Fitness- Assessed using the FITNESSGRAM PACER test. Physical Activity- Measured using the PAQ for adolescents.

There was a positive correlation between fitness (measured as shuttle score) and enjoyment in the control and green exercise conditions (Table 3). Self-reported physical activity was positively correlated with enjoyment in the control condition. This relationship was less strong but remained significant in the green exercise condition (Table 3).

Fitness significantly accounted for 11.2% of the variance in RPE (*β = *−0.37; *P<*0.001), but neither physical activity (*β* = 0.001; *P* = 0.987) or exercise condition (*β = *−0.007; *P = *0.927) made significant contributions. Similarly fitness accounted for a significant (11.7%) proportion of variance in enjoyment scores (*β* = 0.374; *P*<0.001), whilst neither physical activity (*β* = 0.141; *P* = 0.084) or exercise condition (*β* = 0.083; *p* = 0.264) did so.

## Discussion

The aim of this study was to determine if exercising in a natural, ‘green’ environment could have additive effects to the established positive effects that exercise has on children's self-esteem [6]. Despite a reasonable consensus that green exercise can enhance self-esteem in adults [10], [28], this hypothesis has not been examined using rigorous experimental design in children.

The result of our repeated measures experiment showed that green exercise did not create an additional improvement in self-esteem above that observed in the control exercise condition. General physical activity and structured exercise both have positive effects on children's self-esteem [6], [29], however the current findings do not suggest that exposure to nature has a significant additive effect compared to exercise alone. One of the proposed mechanisms for the additive effects of green exercise is the ability of green spaces to provide distraction from daily stresses [11], [13]. Since the current study was performed during a physical education lesson whereby teachers were present and instructing the participants, it is possible that the green run was merely viewed as an extension of school activity. If this was the case, the green run may not have had the same impact as it would have done if it was performed outside of the school day.

The lack of additional impact of green exercise for self-esteem in children might also be due to their low levels of everyday interaction with nature. The current generation of youth spend less time interacting with nature than previous generations [30] due to parental concerns over stranger danger, increasing road traffic [31], [32], the decline in the availability of green space [33], [34] and the attraction of indoor technology [35]. Only 10% of young people have regular contact with nature compared to the 40% of adults who did so 30–40 years ago [35]. Young people are increasingly confined to indoor and urban areas, and may have developed a disconnection from and lack of understanding of the natural world as a consequence [30]. It is proposed that to receive benefits from having contact with nature an individual needs to understand its features and be ‘connected’ to it in some way [36]. Thus it is possible that children are not benefitting from green exercise like adults, because they are not connected to the natural environment in the same way that adults are.

### Assessment of secondary aims

Whilst there was no additional impact of green exercise on self-esteem, there may be other potential benefits to ‘green exercise’ as a means to engage people with exercise and to help adherence to programmes through enjoyment. To assess the other potential benefits of green exercise and to account for individual differences in physical activity and fitness we examined differences in enjoyment and perceived exertion between green and control exercise. We also aimed to explore whether green exercise altered the relationships that measures of physical activity and fitness had with enjoyment and perceived exertion.

There were no significant differences between enjoyment and ratings of perceived exertion in the control and green conditions. While these findings support the null hypothesis this result is noteworthy because the green exercise condition was actually more physically demanding than the control condition, for two reasons. Running over rough terrain requires greater energy expenditure than running on manmade surfaces [37]. The experiment was conducted in the English autumn after heavy rainfall (although conditions were rain-free on all testing days). The children were required to get wet feet, jump large puddles and all finished the course with significant amounts of mud and debris attached to their training shoes. The green condition also had an overall climb (and subsequent descent) of 46 m compared with the completely (±2 m) flat control run. The physical work of carrying body weight means that running uphill requires greater energy expenditure but gradient also negatively affects running economy [38]. Therefore, although we did not measure energy expenditure, we can assume that this was greater in the green condition. The identical RPE and enjoyment scores may give some tentative indication that green exercise feels easier and that natural environments can help more intense exercise to be as enjoyable as less intense exercise in an urban environment. Green exercise may therefore be an important tool for enabling children to achieve activity recommendations and could improve exercise adherence.

We did find significant associations between baseline values for fitness and physical activity and RPE. Unsurprisingly, fitness negatively correlated with RPE; the fitter the child, the easier they perceived the run to be. This was similar in both conditions. Differences in genetic endowment, maturity and sex may all account for differences in fitness. Physical activity is closely related to fitness in children. Unlike fitness, however, it is a modifiable behaviour and a good target for interventions aiming to improve children's health. In the control condition there was a negative association between physical activity and RPE. In common with fitter children, the more active children perceived the control run as easier. This was not the case in the green exercise test. Physical activity was unrelated to perceived exertion meaning that exertion within the green condition was perceived similarly by children with differing levels of habitual physical activity. This may be a potential avenue for future research into green exercise. If less active participants perceive green exercise to be ‘easier’ (due perhaps to distraction) then it may be a means by which to engage those in most need, and with most to potentially gain [39] from exercise.

More active children tend to enjoy formal PE more [16], [17], but it is difficult to determine the direction of this effect. Enjoyment is an important factor mediating engagement in PE [40] and as experience of PE at school can predict adult activity patterns (especially in females) we wanted to know how enjoyment of the two conditions was related to physical activity and aerobic fitness. Unsurprisingly, enjoyment was positively associated with both fitness and physical activity in the control condition. The fitter or more active the child the greater enjoyment rating they gave the control run. There was a similar positive relationship between fitness and enjoyment in the green exercise condition; however the relationship between physical activity and enjoyment was slightly attenuated for the green exercise condition.

Together with the finding from RPE the attenuated association between physical activity and enjoyment during green exercise is encouraging. Compared with a standard 1.5 mile run, the green exercise condition attenuated the well documented association showing that more active children find PE activities less strenuous and was more equally enjoyable for children with a range of activity levels. A study designed with the primary aim of confirming the associations is needed, perhaps also using alternative measures of enjoyment after each exercise condition.

### Limitations and Recommendations

There are several limitations to the present study. The Rosenberg Self-Esteem Scale is typically used as a trait scale and may have reduced the study's sensitivity. This selection was a deliberate attempt to replicate findings of earlier green exercise studies reviewed [10], many of which have successfully used this tool to show changes in self-esteem. Our aim was to test the green exercise hypothesis using a robust scientific design and analysis. We therefore needed to use the same tools as previous researchers. Future studies would benefit from the use of a state self-esteem questionnaire [41].

The control condition, although typical of a school PE lesson in some respects remained somewhat artificial. The time spent exercising was relatively short and the lesson was contaminated by the presence of external researchers. This was the second lesson the researchers had attended as fitness testing and anthropometry were carried out the previous week but we cannot rule out some reactivity of the children to our presence. The condition was also held outside and although the children were contained within an urban environment, they could occasionally see trees or grassy patches at some points during their run. Furthermore, the differences between the two courses in terms of terrain and elevation makes comparing the energy expenditure and ‘effort’ needed to complete them difficult. This is, however, a common weakness in such natural experiments and was unavoidable in the present protocol. It would however be useful for future research to obtain a measure of energy expenditure so that exercise conditions can be compared. It would also be beneficial to perform a follow-up study incorporating different green exercise modes such as walking and running, in order to determine if the role of the environment in improving self-esteem varies according to exercise intensity. Factors such as relatedness to nature, perceptions and attitudes and perceived level of engagement would also need to be controlled for to develop further insights into the effects of green exercise in children. The school itself was chosen because of its location and was unique of nine potential schools visited in being able to offer both an urban setting and a nearby, wooded, rural area in which to exercise.

The use of retrospective RPE has been mentioned previously and future studies should take such measures during the exercise trails. To further examine the relationships activity and fitness may have with enjoyment and RPE during green exercise a larger sample is needed. This would also allow the observation of between-sex differences. We could not make relevant comparisons here due to sample size but of note, all changes and correlations were similar in magnitude between boys and girls, despite lower fitness and physical activity in the latter.

### Conclusions

This study assessed the impact of green exercise using a rigorous methodology in children. The effects of green exercise on children's self-esteem were no greater than those of exercise using standard analysis. Despite no evidence for synergy between exercise and nature for increasing self-esteem we did find some promising trends for both enjoyment and ratings of perceived exertion. Though identical between the conditions the increased energy expenditure during the green condition may indicate that natural environments make more intense exercise seem equally as easy and enjoyable as less intense exercise in built environments. Green exercise could therefore be used as a tool for engaging children in more moderate to vigorous physical activity, in line with current recommendations. Also promising was the lack of association between physical activity and perceived exertion and the attenuated association between physical activity and enjoyment in the green condition. We tentatively suggest that green exercise may offer an enjoyable and accessible form of physical activity to less active children who may not typically be well-engaged in physical education.
